# Neuregulin3 alters cell fate in the epidermis and mammary gland

**DOI:** 10.1186/1471-213X-7-105

**Published:** 2007-09-19

**Authors:** Heena Panchal, Olivia Wansbury, Suzanne Parry, Alan Ashworth, Beatrice Howard

**Affiliations:** 1 The Breakthrough Breast Cancer Research Centre, Institute of Cancer Research 237 Fulham Road, London SW3 6JB, UK

## Abstract

**Background:**

The Neuregulin family of ligands and their receptors, the Erbb tyrosine kinases, have important roles in epidermal and mammary gland development as well as during carcinogenesis. Previously, we demonstrated that Neuregulin3 (Nrg3) is a specification signal for mammary placode formation in mice. Nrg3 is a growth factor, which binds and activates Erbb4, a receptor tyrosine kinase that regulates cell proliferation and differentiation. To understand the role of Neuregulin3 in epidermal morphogenesis, we have developed a transgenic mouse model that expresses Nrg3 throughout the basal layer (progenitor/stem cell compartment) of mouse epidermis and the outer root sheath of developing hair follicles.

**Results:**

Transgenic females formed supernumerary nipples and mammary glands along and adjacent to the mammary line providing strong evidence that Nrg3 has a role in the initiation of mammary placodes along the body axis. In addition, alterations in morphogenesis and differentiation of other epidermal appendages were observed, including the hair follicles. The transgenic epidermis is hyperplastic with excessive sebaceous differentiation and shows striking similarities to mouse models in which c-Myc is activated in the basal layer including decreased expression levels of the adhesion receptors, α6-integrin and β1-integrin.

**Conclusion:**

These results indicate that the epidermis is sensitive to Nrg3 signaling, and that this growth factor can regulate cell fate of pluripotent epidermal cell populations including that of the mammary gland. Nrg3 appears to act, in part, by inducing c-Myc, altering the proliferation and adhesion properties of the basal epidermis, and may promote exit from the stem cell compartment. The results we describe provide significant insight into how growth factors, such as Nrg3, regulate epidermal homeostasis by influencing the balance between stem cell renewal, lineage selection and differentiation.

## Background

Neuregulins are a family of ligands that signal through the four Erbb receptor tyrosine kinases to activate pathways. This network mediates a wide range of processes that have relevance to both developmental processes and cancer, including cell adhesion, differentiation, proliferation, migration and death [1]. Our previous studies suggested that Nrg3 signaling acts to promote the initiation of mammary placode development [2]. The cognate receptor for Nrg3, Erbb4, is required for terminal differentiation of the mammary gland and lactation fails in its absence [3,4]. Impaired mammary epithelial proliferation and lobuloalveolar defects are also observed in *Nrg1α*-null mice [5]. Analysis of *Amphiregulin*-null mice demonstrated the requirement of *Amphiregulin *for ductal outgrowth at puberty [6]. Studies of compound null mutations for genes for the Egf-related ligands (*Amphiregulin, Egf, and Tgfα*) demonstrated failed lactation due to abnormal alveolar development and differentiation, whereas no lactation defect is apparent when these genes are singly mutated [6]. Studies of mammary tissue from *Erbb1 *and *Erbb2*-null mouse models have shown that these genes have important roles in mammary ductal outgrowth/morphogenesis [7-10]. Mouse models which overexpress the ligand, Nrg1, or the Erbb2 receptor (both wild-type and activated forms) display severe hyperplastic epidermal and/or mammary phenotypes depending on the cell type specificity of promoter used to drive transgene expression [11-15]. These mouse models have provided a useful framework for understanding how this ligand/receptor network acts to promote differentiation, outgrowth, and carcinogenesis of epithelial tissues.

Egf and Egf-like ligands that signal through Egfr were initially discovered and named due to their profound effects on epidermal development [16-18]. Egf increases epidermal thickness and cellularity and stimulates proliferation of epidermal keratinocytes [16]. Mice with targeted disruptions of *Egfr/Erbb1 *are usually embryonically lethal but this is strain-dependent, so it is possible to analyze hypomorphs, and viable strains display epithelial hypoplasia [19-21]. Mice harbouring the *Waved-2 *allele of *Egfr *develop abnormal hair that appears wavy and also display a mild lactation defect [22]. Other Egf-like ligands expressed by keratinocytes include Amphiregulin [23], Betacellulin [24], Heparin-binding Egf-like growth factor [25], and TGFα [26]. Using wound healing models, Nrg1/Heregulin (HRG) has been implicated in epithelial migration and differentiation [27]. The Nrg1 isoforms, HRGα and HRGβ, elicit different effects in cultured keratinocytes [28]; HRGα acts as a potent motility factor whereas HRGβ has no effect on migration in wound healing assays [27,28]. In contrast, in normal melanocytes, HRGβ significantly enhances cell migration, but not proliferation, while HRGα has no effect on migration or cell growth of normal melanocytes [29]. Despite the profound biological effects that Egf-like ligands elicit, their modes of action are not yet fully understood, particularly with respect to their ability to regulate cell fate and lineage commitment.

Mammary placodes are thought to arise as a result of local cell migration [30] and early stages are characterized by epithelial stratification [31,32]. Our previous studies indicated that Nrg3 promotes the differentiation of squamous epithelia into mammary epithelia [2]. In the mouse embryo, Nrg3 appears to regulate epithelial stratification or local epithelial aggregations at the sites that mammary placodes will form. Mice harbouring the *ska *mutation, a hypomorphic allele of *Nrg3*, often fail to form placode three; this can be restored after culture with recombinant Nrg3-Egf. Placode morphogenesis is governed by molecules that alter cell adhesion dynamics and, in some cases, proliferation of pluripotent epithelial cells [33,34]. In the mammary gland, as with the development of all epidermal appendages, undifferentiated stem cells are committed to specific lineages and a population of cells with a high capacity for proliferation is delimited, which subsequently differentiates [33,35,36]. The role of Nrg3 signaling in the determination of epidermal stem cell fate remains largely uncharacterized. Therefore, we developed a mouse model to examine the outcome of ectopic expression of *Nrg3 *in the basal layer of the developing epithelia under the control of the Keratin14 (K14) promoter. This promoter results in expression beyond the normal spatial and temporal domains of Nrg3 expression into the stem cell compartment. Expression of Nrg3 in the basal layer of the epidermis resulted in alterations in a variety of epidermal organs including the skin, hair follicles, sebaceous glands, external genitalia and mammary glands. Analysis of epithelial differentiation and progenitor markers suggests that differentiation of progenitor cell populations is profoundly altered by *Nrg3 *expression.

## Results

### Nrg3 is expressed in the developing epidermis and hair follicles

**Figure 1:**
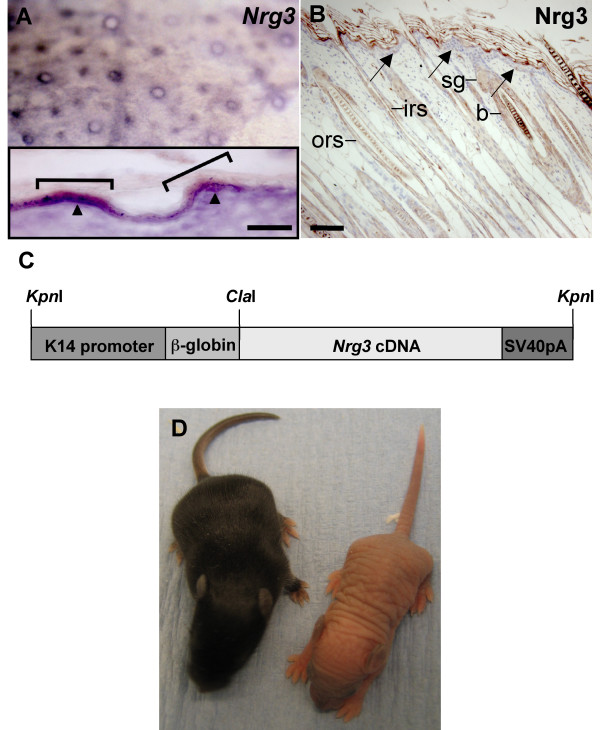
**Endogenous expression pattern of *Nrg3*, K14-*Nrg3 *transgene, and postnatal phenotype of a K14-*Nrg3 *founder mouse**. (**A**) *Nrg3 *is expressed in the developing hair follicles at E14.5. Inset shows frozen section of the whole mount which confirmed the localization of *Nrg3 *in the nascent hair follicles (brackets). Arrowheads indicate intense basal epithelial expression. Suprabasal and diffuse mesenchymal *Nrg3 *expression are also observed. The scale bar represents 100 μm. (**B**) Nrg3 is expressed in the granular and cornified layers and in a few basal cells (arrows) of the postnatal epidermis and at low to moderate levels in the hair follicles in the outer root sheath and inner root sheath at P7. The scale bar represents 50 μm in (B). (**C**) Transgene used for ectopic expression of *Nrg3*. The transgene includes a human K14 promoter, a rabbit β-globin intron and an SV40 polyadenylation site. (**D**) Phenotype of one K14-*Nrg3 *founder at P7. Mutant mice are hairless and have thick, pale and wrinkled skin. A non-transgenic littermate is on the left. b, bulge; irs, inner root sheath; ors, outer root sheath; sg, sebaceous gland.

We have shown previously, using both *in situ *hybridization and immunohistochemistry, that Nrg3 is expressed in the developing embryonic mouse epidermis from E12-E13 [2]. *Nrg3 *is also expressed in developing hair follicles at E14.5 (Fig. 1A). To investigate Nrg3 expression in postnatal skin, we used immunohistochemistry on both frozen and fixed mouse epidermis (Fig. 1B and data not shown). We found that Nrg3 is expressed in the granular and cornified layers and in very few cells of the basal layer of the postnatal interfollicular epidermis, and at low to moderate levels in the hair follicles along the outer root sheath including the bulge and along the inner root sheath (Fig. 1B and Additional File 1).

### Ectopic expression of Nrg3 in the epidermis causes epidermal defects and early lethality

To extend the temporal and spatial range of *Nrg3 *expression in the epidermis, and determine whether Nrg3 can modulate the development of epidermis and its appendages, we generated transgenic mice expressing full-length *Nrg3 *from the human K14 promoter (Fig. 1C). The K14 promoter is expressed in the periderm starting at E9.5 and, once stratified, in the basal layer (progenitor/stem cell compartment) of mouse epidermis and the outer root sheath of the hair follicles [37]. Founder mice were produced by pronuclear injection of the linearized transgene into fertilized eggs (Additional File 2). Levels of transgene expression in the epidermis of mice were determined by immunohistochemistry or by *in situ *hybridization (data not shown). Six male K14-*Nrg3 *transgenic founders exhibited a similar thickened, wrinkled, hairless phenotype that encompassed the entire skin of the animal (Fig. 1D). Of these, four died a few days after birth or were unwell and were culled. However, two male founders expressing lower transgene levels were viable and exhibited hyperplastic epithelial phenotypes but did not display other epidermal appendage phenotypes, or the phenotypes were less severe. Furthermore, two male founder mice that were mosaic for transgene levels expressed variable levels of transgene expression and were also viable.

### Histopathology of K14-*Nrg3 *transgenic skin

**Figure 2:**
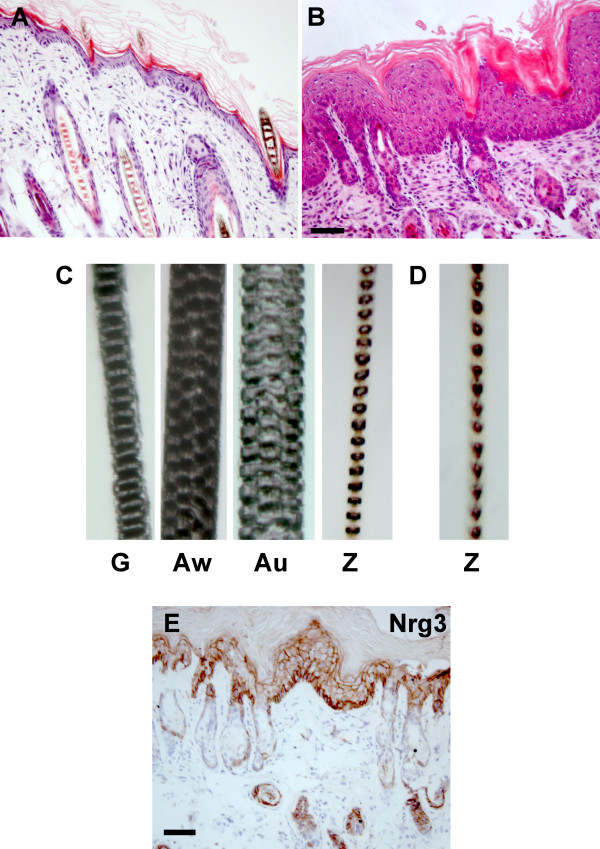
**K14-*Nrg3 *transgenic mice display hyperplastic epithelia and hair follicleab normalities**. Histological analysis of dorsal skin from K14-*Nrg3 *and age-matched non-transgenic littermate. (**A**) Skin section stained with H&E from non-transgenic littermate at P7. (**B**) Skin section stained with H&E from K14-*Nrg3 *transgenic founder at P7. The transgenic epidermis shows hyperplasia and enlargement of cells and nuclei compared with non-transgenic littermates. Expanded suprabasal, granular and cornified layers are present in the interfollicular epidermis (IFE). A large infiltration of fibroblasts was observed in the transgenic skin. Hair follicles and sebaceous glands were also hyperplastic. (**C**) Four hair shaft types are present in a non-transgenic littermate.(**D**) Only one hair shaft type (zigzag) is present in a lower *Nrg3 *expressing K14-*Nrg3 *transgenic line that did form some sparse wavy hair. (**E**) Transgenic expression pattern of Nrg3 from K14-*Nrg3 *transgenic skin at P7. Nrg3 is expressed in the basal, suprabasal, and granular layers of the epithelium and in the outer root sheath (ORS) of hair follicles.(G, guard; Au, Auchene; Aw, Awl; Z, zigzag) The scale bar represents 50 μm.

Histological examination of seven independent K14-*Nrg3 *transgenic founders revealed that the skin shared several unusual features. The epidermis displayed expanded suprabasal, granular and cornified layers when compared to non-transgenic littermates (Fig. 2A, B). Nucleated cells were present throughout the epidermis, even in the later differentiated compartments, except for the cornified layers, which were anucleate. An increase in the cellular density of the dermis was also observed. A large infiltration of fibroblasts was apparent and the fat layer appeared much thinner in the transgenic skin. Hair follicles were hyperplastic and mis-orientated within the dermis and sebaceous glands were mis-positioned and hyperplastic. All components of the hair follicle are present and differentiation proceeds normally but is disrupted at a very late stage so that the hair fiber either fails to form or when a fiber does form it does not penetrate through the thickened epithelium. However, mice derived from one lower expressing K14-*Nrg3 *transgenic line did form some sparse wavy hair but only one hair type (zigzag) was present compared to the four hair types normally found in non-transgenic littermates (Fig. 2C, D).

Using an antibody to the Egf domain of Nrg3, we detected Nrg3 expression in the basal, suprabasal, and granular layers of the epithelium and in the outer root sheath of hair follicles in the transgenic founder lines (Fig. 2E). Cytoplasmic and membrane staining is observed suggesting that both membrane-spanning and secreted forms of Nrg3 are produced in the transgenic K14-*Nrg3 *mice, as expected from the isoform of Nrg3 present in the K14-*Nrg3 *construct. Non-transgenic littermates express Nrg3 in a few basal and in the granular and cornified layers and low to moderate levels throughout the hair follicles (Fig. 1B).

### Altered Erbb receptor activation is observed in K14-*Nrg3 *transgenic skin

**Figure 3:**
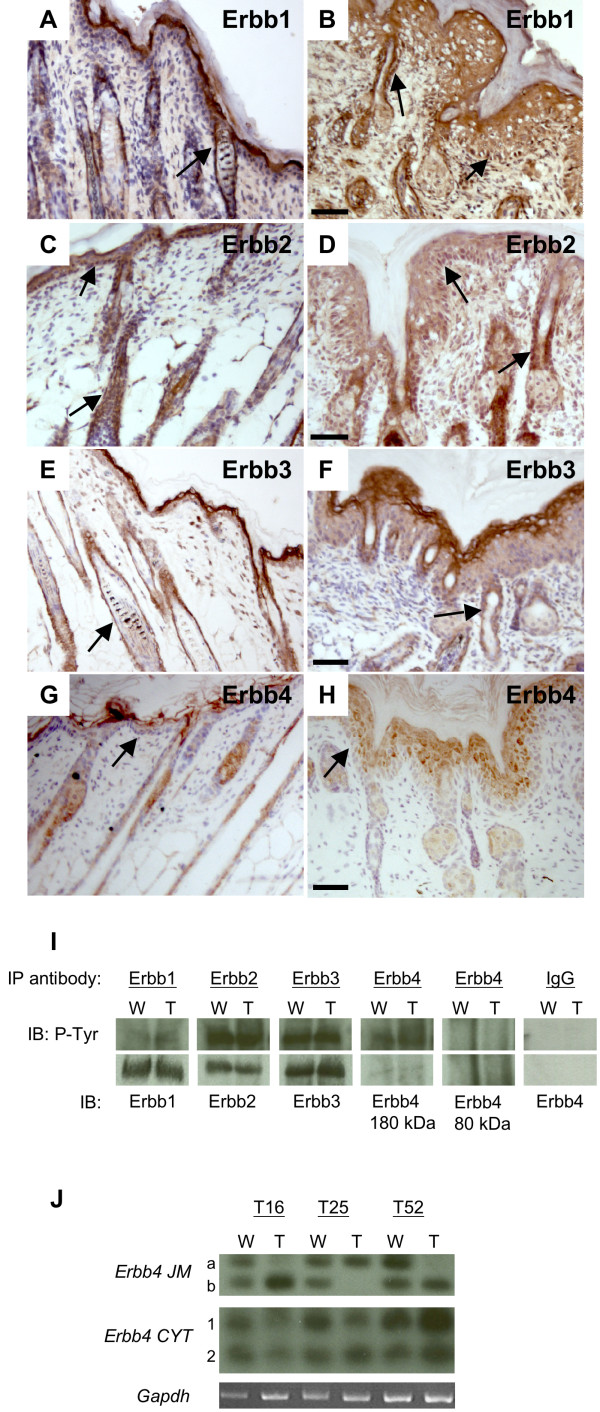
**Expression and activation of Erbb receptors in dorsal epidermis from non-transgenic and K14-*Nrg3 *transgenic littermates at P7**. (**A**, **C**, **E**, **G**) Skin from non-transgenic littermate. (**B**, **D**, **F**, **H**) Skin from K14-*Nrg3 *transgenic founder. (**A **and **B**) Erbb1 is expressed on the cell membranes throughout the IFE and ORS. (**C **and **D**) Erbb2 is weakly expressed on the cell membranes throughout the IFE and ORS. (**E **and **F**) Erbb3 is expressed on the cell membranes throughout the IFE and ORS and is more highly expressed in the granular layers. (**G **and **H**) Erbb4 is expressed at very low levels in the IFE and at low levels in the hair follicles of a non-transgenic littermate. Erbb4 is expressed at increased levels in K14- *Nrg3 *transgenic skin throughout the differentiating layers of the IFE and in the ORS (mainly cytosolic and weak membranous). Basal expression of receptor in the IFE and ORS is denoted by arrows in A-H. The scale bar represents 50 μm. (**I**) Immunoprecipitation and immunoblot analysis of tyrosine phosphorylation of Erbb1, Erbb2, Erbb3, or Erbb4 immunoprecipitates from dorsal non-transgenic or K14-*Nrg3 *transgenic skin. Each of the four Erbbs was immunoprecipitated (IP) from 1 mg of lysate and analyzed by immunoblotting (IB) with anti-phosphotyrosine (P-Tyr). The filters were stripped and reprobed with receptor-specific antibodies to determine receptor levels. W denotes wild-type non-transgenic skin sample and T denotes transgenic skin sample.(**J**) *Erbb4 *isoforms expressed in the epidermis. *JM and CYT Erbb4 *isoform levels as determined by semi-quantitative RT-PCR. Dorsal skin displays similar levels of expression of *CYT-1 *and *CYT-2 *levels in non-transgenic and K14-*Nrg3 *transgenic epidermis. Differential expression of *JM-a *and *JM-b *levels were observed such that one of the isoforms is preferentially expressed in the K14-*Nrg3 *transgenic epidermis (T) compared with that isolated from non-transgenic littermates (W). Three independent transgenic lines (T16, T25, T52) were analyzed and compared with three non-transgenic siblings. *Gapdh *was amplified in parallel as a control.

Erbb1, Erbb2, and Erbb3 expression has been reported in both human and mouse skin [15,38,39]. Erbb4 expression has not been reported in postnatal mouse epidermis although expression has been observed in both embryonic and adult human skin [38]. *Erbb4 *is expressed in the epithelia of the developing mammary placode from the time it is morphologically distinct [2]. We examined expression of the four Erbb receptors in both transgenic and non-transgenic postnatal skin. Erbb1 and Erbb2 are expressed in the basal epithelia and differentiating epidermal layers and in the outer root sheath of the hair follicles of both non-transgenic and transgenic skin (Fig. 3A–D). Erbb3 is expressed in the basal, suprabasal layers and at higher levels in the granular epithelial layers and in the outer root sheath of the hair follicles of both transgenic and non-transgenic skin (Fig. 3E, F). In non-transgenic mouse epidermis, we detect only very low levels of Erbb4 in the interfollicular epidermis and we observed low levels of expression in the hair follicles (Fig. 3G and Additional File 1). However, in K14-*Nrg3 *transgenic skin, we observed significantly increased levels of Erbb4 in the epidermis (mainly cytosolic and weak membranous) (Fig. 3H), which suggest that Nrg3 expression in the basal epithelia might induce an autocrine signaling loop.

We explored the signaling events that mediate the Nrg3 response in the epidermis. Erbb1 is slightly more activated in the transgenic epidermis when compared to the non-transgenic epidermis (Fig. 3I). Both Erbb2 and Erbb3 activation levels are similar in the transgenic and non-transgenic epidermis (Fig. 3I). Ectopic Nrg3 promotes a slight increase in the levels of Erbb4 and a moderate increase in tyrosine phosphorylation of Erbb4 in the transgenic epidermis (Fig. 3I). The possible active signaling complexes in the transgenic epidermis are therefore Erbb1:1, Erbb1:2, Erbb1:3, Erbb2:3, Erbb2:4, Erbb4:4 since Erbb2 requires a Neuregulin binding receptor to signal and Erbb3 homodimers are inactive.

Signaling through the Erbb4 receptor is complicated as four major variants of the receptor exist [40]. Alternative splice variants of *Erbb4 *are produced at two distinct sites resulting in four possible isoforms of the receptor. JM-a and JM-b variants encode variable extracellular juxtamembrane isoforms that can be cleaved by matrix metalloproteases (MMPs) (JM-a) or are resistant to such cleavage (JM-b) [41]. Two cytoplasmic variants also exist which contain (CYT-1) or lack (CYT-2) an exon corresponding to sixteen amino acids that encodes a phosphatidylinositol 3-kinase (PI3K) docking site [42]. We examined the expression of *Erbb4 *isoforms in the epidermis of three independent K14-*Nrg3 *transgenic lines and compared them to the epidermis from non-transgenic siblings. *CYT-1 *and *CYT-2 *isoforms were expressed at similar levels in both the transgenic and non-transgenic epidermis (Fig. 3J). We found that unusual *Erbb4 JM *isoform profiles exist in the transgenic epidermis that are distinct from those expressed in non-transgenic epidermis. Similar levels of the *JM-a *and *JM-b* isoforms are expressed in non-transgenic epidermis. We observed deviations such that either *JM-a *or *JM-b *isoforms were preferentially expressed in the transgenic lines (Fig. 3J). These results suggest that misexpressing *Nrg3 *in the epidermis alters the ratios of *Erbb4 JM *splice isoform gene expression.

The JM-a domain of *Erbb4 *encodes a cleavable receptor which can transmit signals to the cytoplasm and nucleus [43-45]. Binding of Nrg1 to the Erbb4 JM-a ectodomain isoform results in the cleavage and shedding of the 120 kD ectodomain. This is mediated by tumor necrosis factor-alpha converting enzyme and results in the production of an 80 kD intracellular membrane-bound domain [46]. This domain can then be cleaved by gamma-secretase to release an 80 kD intracellular domain [47]. Immunoprecipitate analysis of Erbb4 receptor signaling detected no activated 80 kD isoform in the epidermis from transgenic or non-transgenic littermates (Fig. 3I). Therefore, activated Erbb4 exists predominantly in the 180 kD membrane-bound isoform in the epidermis of both transgenic and non-transgenic littermates (Fig. 3I).

### K14-*Nrg3 *transgenic mice exhibit other epithelial appendage phenotypes

We observed fewer K14-*Nrg3 *transgenic females than predicted and the overall efficiency of transgenesis was low (Additional File 3), suggesting that ectopic expression of *Nrg3 *in the epidermis during embryogenesis may be partially lethal, particularly in females. Four female transgenic founders with obvious skin phenotypes were obtained. Of these, two were found dead shortly after birth. Histological analysis showed a skin phenotype similar to that observed in the other male transgenic founders (data not shown). The other two female founders both displayed a hairless, wrinkled, thickened, pale skin similar to that observed in the male founders except this was present on approximately half of the body of the mouse, consistent with integration of the transgene at the two-cell stage (Additional File 4).

**Figure 4:**
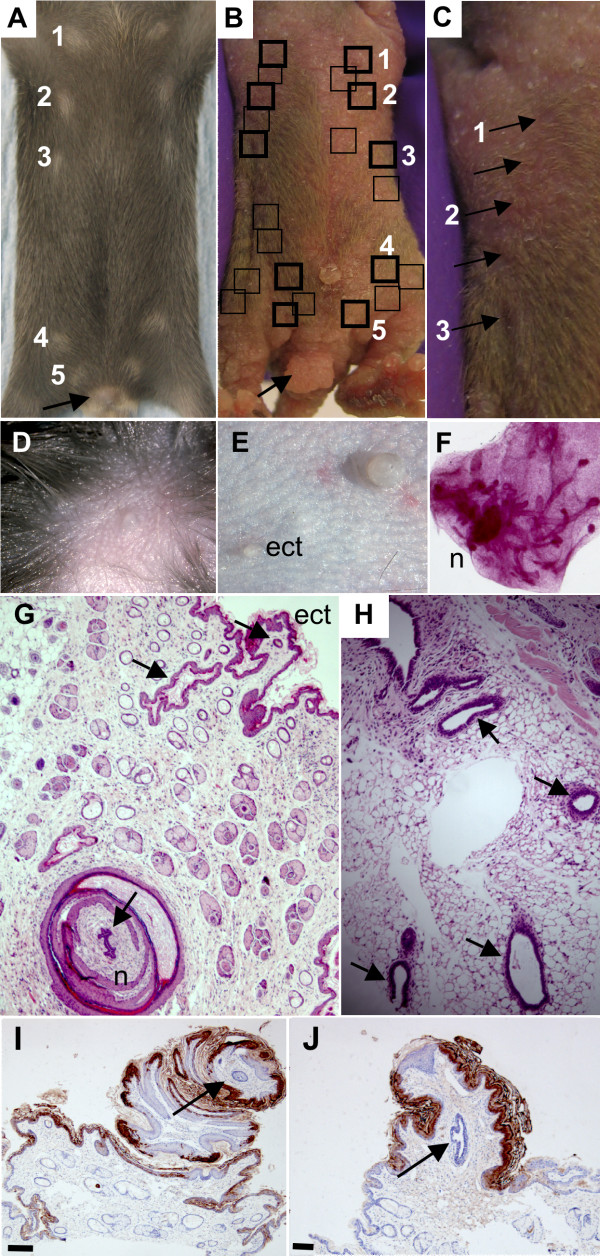
**Ectopic expression of *Nrg3 *in the epidermis results in the formation of ectopic nipples and mammary glands**. (**A**) Ventral surface of non-transgenic littermate at P12 with the five pairs of nipples marked. Numbers denote the positions of the five normal nipples. External genitalia of female non-transgenic littermate is indicated with an arrow. (**B**) Ventral surface of one chimeric founder female at P12 with the five pairs of endogenous nipples boxed in bold, and ectopic nipples are marked with black boxes. Numbers denote the positions of the five normal nipples. Enlarged external genitalia of K14-*Nrg3 *female is indicated with an arrow. (**C**) Magnification of left side of (B) showing close-up of nipples which are denoted by arrows. Numbers denote the position of the endogenous nipples 1–3. (**D**) Non-transgenic nipples are small and round. Image acquired at 160× magnification.(**E**) Transgenic K14-*Nrg3 *nipples are very large and round. Ectopic nipples are usually smaller than the endogenous transgenic nipples and of a similar size to non-transgenic nipples. Ectopic nipple is indicated (ect). Image acquired at 160× magnification. (**F**) Carmine-stained whole-mount of mammary fat pad associated with ectopic nipple showing nipple (n) connected to a mammary ductal tree.(**G**) H&E-stained section of ectopic nipple showing associated mammary epithelial tissue. Endogenous nipple (n) and ectopic nipple (ect) are indicated and associated epithelial ducts are denoted by arrows. (**H**) H&E-stained section of fat pad and mammary ducts (indicated with arrows) associated with an ectopic nipple (shown in (G)). (**I**) K2e, a marker of nipple skin, is expressed in the endogenous K14-*Nrg3 *nipple skin. The primary mammary duct is indicated by an arrow. The scale bar represents 50 μm. (**J**) K2e, a marker of nipple skin, is expressed in the ectopic K14-*Nrg3 *nipple skin. The primary mammary duct is indicated by an arrow. The scale bar represents 50 μm.

At P12, these two female founders were scored for the number and placement of nipples on the ventral surface. In both females, in addition to the 5 pairs in the normal location (Fig. 4A), there were multiple ectopic nipples both along the "mammary line" and also in adjacent locations (Fig. 4B, C). Results from breeding from these females were consistent with chimerism. The one transgenic male pup produced in the first litter displayed the same hairless, wrinkled, thickened, pale skin phenotype (over its entire body surface) shared by the other transgenic founder males and died at P5. The female founder mice were culled to collect tissue samples for analysis and verify that mammary tissue (fat pad and epithelial ducts) was associated with the ectopic nipples (Fig. 4E, F, G, H). Ectopic nipples observed on transgenic mice are usually smaller than the endogenous nipples which are very large when compared to non-transgenic nipples (Fig. 4D, E) but varied considerably in size and are sometimes as large as the endogenous transgenic nipples (Fig. 4I, J). Both endogenous and ectopic nipples express Keratin2e, a nipple marker for mouse nipple epidermis [48] (Fig. 4I, J).

**Figure 5:**
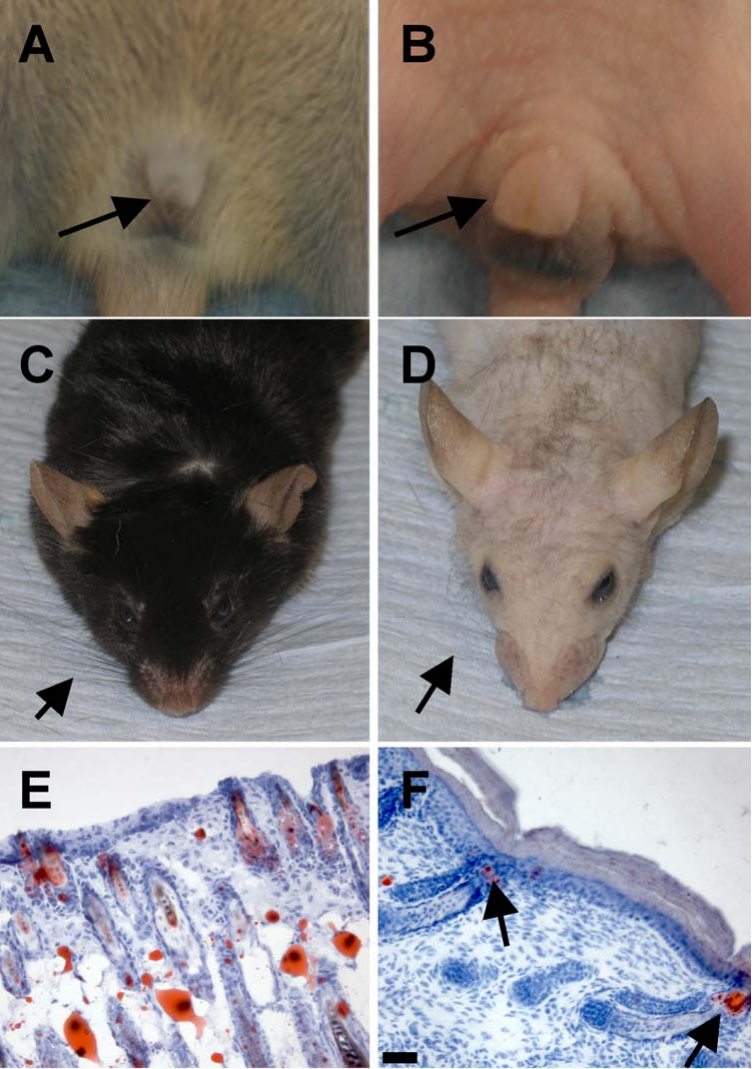
**Ectopic expression of *Nrg3 *in the epidermis causes defects in the external genitalia, vibrissae, and hair**. (**A**) External genitalia of male non-transgenic littermate is indicated with an arrow. (**B**) Enlarged external genitalia of K14-*Nrg3 *transgenic male is indicated with an arrow. (**C**) Vibrissae of non-transgenic littermate are indicated with an arrow. Note normal fur growth. (**D**) Vibrissae of K14-*Nrg3 *transgenic mouse (indicated with an arrow) are sparse and wispy in comparison to non-transgenic littermate. In one K14-*Nrg3 *founder and most progeny from this line sparse fur appeared as the mice aged (usually from P100 onward). (**E**) Oil red O -stained section of skin from non-transgenic littermate at P7 showing normal distribution of hair follicles. (**F**) Oil red O -stained section of skin from K14-*Nrg3 *transgenic founder at P7 showing abnormally close juxtaposition of hair follicles which are indicated with arrows.

Both male and female mice exhibited enlarged external genitalia when compared to non-transgenic littermates (Fig. 4A, B and Fig. 5A, B). Despite the increased size of the external genitalia, most mice were fertile and could breed. Most transgenic founders displayed very sparse and wispy whiskers and one line eventually grew sparse and wavy hair (Fig. 5C, D). Clusters of hair follicles were observed in some skin sections, which is indicative of abnormal patterning events (Fig. 5E, F). One transgenic founder line exhibited premature tooth eruption (culled at P7 as it could no longer suckle) but normal tooth number and cusp patterning were observed in these and other transgenic founder lines (data not shown).

### Hyperproliferative marker profile but a normal terminal differentiation profile observed in K14-*Nrg3 *transgenic interfollicular epidermis

**Figure 6:**
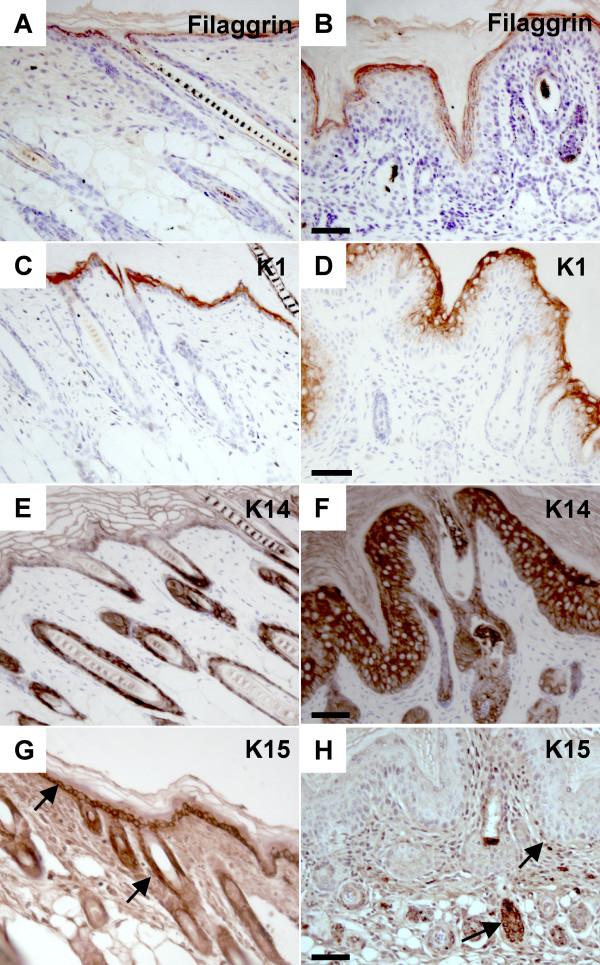
**Immunohistochemistry using epidermal differentiation markers**. Immunohistochemistry of sections of dorsal skin from transgenic mice and non-transgenic littermates at P7 with antibodies to the proteins indicated. (**A **and **B**) Expression of Filaggrin, a terminal differentiation marker, is maintained in the granular and cornified layers of non-transgenic (A) and K14-*Nrg3 *transgenic (B) skin. (**C **and **D**) K1, a marker for committed early differentiating cells, is expressed in the suprabasal layer of non-transgenic (C) and K14-*Nrg3 *transgenic (D) skin. (**E **and **F**) K14, a marker for basal proliferating keratinocytes, is expressed in the basal layer of non-transgenic skin and the ORS of the hair follicles (E) and is expressed at high levels throughout the basal, suprabasal, granular and cornified layers of K14-*Nrg3 *transgenic skin and ORS of the hair follicles (F). (**G **and **H**) Expression of K15 is normally restricted to basal keratinocytes as observed in non-transgenic littermates (G) and marks undifferentiated cells with low turn over. Expression of K15 is reduced in the basal IFE in K14-*Nrg3 *basal epidermis (H). K15 is expressed in the ORS, where it marks the bulge region of the hair follicle, in both normal and mutant epidermis (G and H). Basal expression of K15 in the IFE and ORS is denoted by arrows. The scale bar represents 50 μm.

The status of epidermal differentiation was assessed using immunohistochemistry of paraffin sections. Although the epidermis is thicker in K14-*Nrg3 *skin compared to non-transgenic littermates, markers of terminal epidermal differentiation were present. The distribution of Filaggrin, a terminal differentiation marker, is maintained in the granular and cornified layers (Fig. 6A, B). Similarly Involucrin, another terminal differentiation marker is also expressed in the granular and cornified layers (Additional File 5). The distribution of K1 and K10, markers for committed early differentiating cells, are unaltered and remain in the suprabasal layer (Fig. 6C, D; Additional File 5). Neoplastic skin lesions generally display reduced K1 expression so the transgenic epidermis does not appear to be neoplastic. K5 and K14 were used as markers for the basal proliferating keratinocytes and showed markedly altered expression. Both were expressed at high levels throughout the basal and suprabasal layers of K14-*Nrg3 *transgenic skin (Fig. 6E, F; Additional File 5). Another basal keratin, K15 is normally restricted to basal keratinocytes as observed in non-transgenic littermates (Fig. 6G) and marks undifferentiated cells with low turnover [49]. K15 expression is reduced in the basal interfollicular epidermis in K14-*Nrg3 *basal epidermis (Fig. 6H). K15 expression is retained in the outer root sheath where it marks the bulge region of the hair follicle in both normal and mutant epidermis (Fig. 6G, H) [50]. K15 expression is not compatible with keratinocyte activation and is frequently downregulated in hyperproliferative conditions [51]. The large expansion of suprabasal and granular layers observed in transgenic skin is also a feature of hyperproliferating conditions. Ectopic expression of Nrg3 in the basal layer deregulates the expression pattern of keratin markers, K5, K14, and K15 to one associated with hyperproliferation conditions. The terminal differentiation program of keratinocytes in K14-*Nrg3 *transgenic skin is essentially normal and characterized by excessive production of terminally differentiated cells.

### Nrg3 signaling stimulates epidermal proliferation

**Figure 7:**
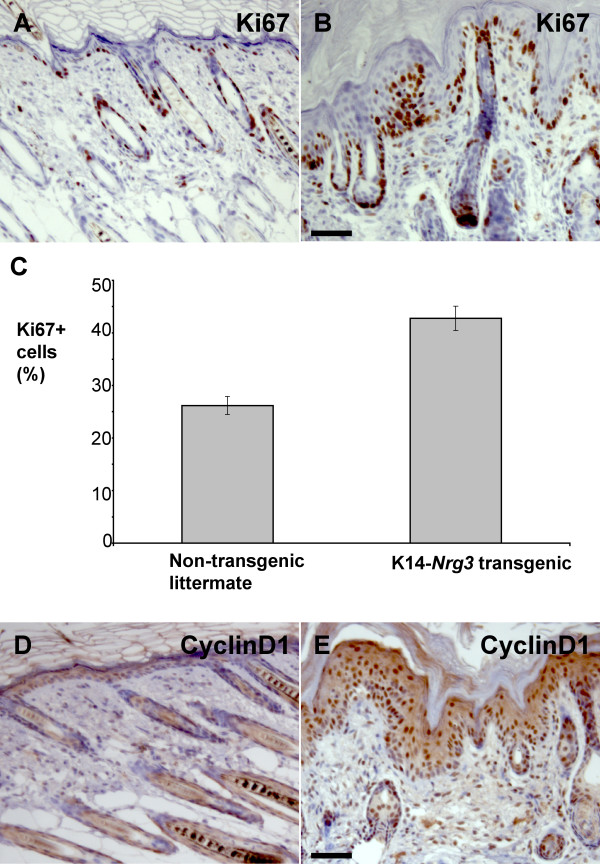
**Increased proliferation in K14-*Nrg3 *epidermis**. Immunohistochemical analysis shows an increase in Ki67 and CyclinD1 in the epidermis. (**A **and **B**) In non-transgenic epidermis (A), Ki67 is restricted to some basal cells. In K14-*Nrg3 *skin (B), Ki67 is expressed throughout the basal layer and in three to four suprabasal layers.(**C**) Quantification of immunohistochemical analysis with Ki67 antibodies. 500 cells were counted. Error bars indicate the standard deviation. The differences are significant between non-transgenic and K14-*Nrg3 *epidermis (p = 0.0176) using the paired student test. (**D **and **E**) In non-transgenic epidermis (D), CyclinD1 expression is restricted to some basal cells. In K14-*Nrg3 *skin (E), CyclinD1 is expressed throughout most of the IFE. The scale bar represents 50 μm.

The increased thickness of the epidermis suggested that Nrg3 stimulates proliferation. Ki67 was used as a proliferation marker for cells (expressed by cells in late G1, S, G2, and M phases). In contrast to normal skin (Fig. 7A), the K14-*Nrg3 *basal cells are nearly all Ki67+ and many suprabasal cells are Ki67+ (Fig. 7B). In one K14-*Nrg3 *transgenic line, approximately 43% of the interfollicular epithelial cells are Ki67+ compared to 26% observed in non-transgenic littermates in postnatal day 7 skin (Fig. 7C). Normally, a restricted number of basal cells express CyclinD1 (which is expressed by cells in G1 phase) (Fig. 7D). However, CyclinD1 expression is observed in the basal, suprabasal, and granular cell layers of K14-*Nrg3 *epidermis (Fig. 7E). Therefore, constitutive expression of *Nrg3 *increases the number of cycling cells in the epidermis, including the basal layer.

### Sustained Nrg3 signaling stimulates sebocyte differentiation

**Figure 8:**
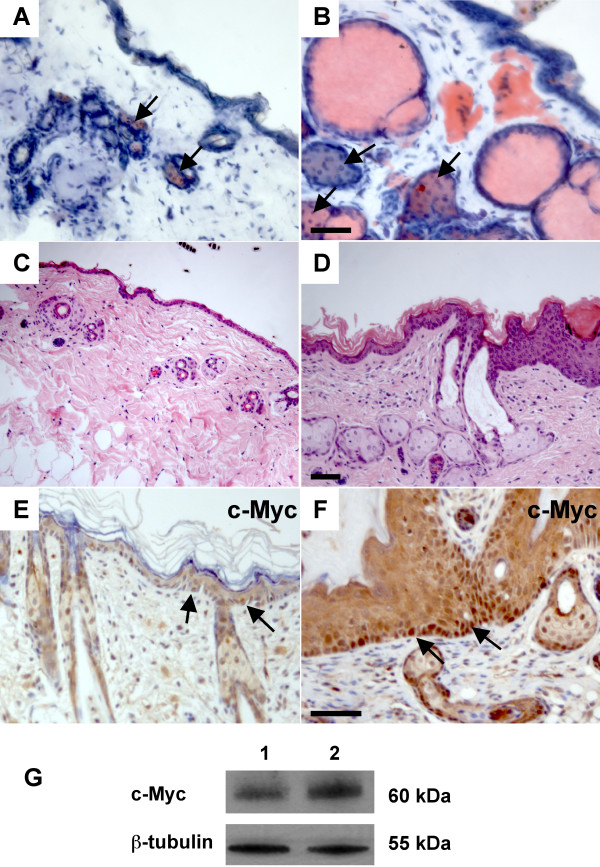
**Nrg3 signaling stimulates sebocyte differentiation**. (**A **and **B**) In non-transgenic epidermis (A), Oil Red O stains the sebaceous gland (indicated by arrows) associated with each hair follicle. In K14-*Nrg3 *skin (B), the sebaceous glands (indicated by arrows) are enlarged. (**C**) Section stained with H&E from non-transgenic littermate at P52. (**D**) Section stained with H&E from K14-*Nrg3 *transgenic skin at P52. Note the excessive sebaceous differentiation. (**E **and **F**) In non-transgenic epidermis (E), c-Myc expression is restricted to a few basal cells (indicated by arrows). In K14-*Nrg3 *skin (F), it is expressed throughout most of the basal epidermis (indicated by arrows). (**G**) Western blot for c-Myc. Lysates were prepared from ventral skin of non-transgenic (lane 1) and K14-*Nrg3 *littermates (lane 2) and blotted with antibodies against c-Myc and β-Tubulin (loading control). The scale bar represents 50 μm.

To examine the differentiation of sebaceous glands, we stained transgenic and non-transgenic skin with Oil Red O, which marks lipid- containing cells, including sebocytes. In normal skin, sebaceous glands were associated with the upper part of each hair follicle (Fig. 8A). In K14-*Nrg3 *transgenic skin, the number and size of sebocytes was greatly increased (Fig. 8B). This is especially dramatic in skin samples collected from older mice, where essentially all of the abnormal hair follicles are filled with sebocytes and only few structures resembling hair follicles remain (Fig. 8D). This suggests that Nrg3 signaling stimulates sebocyte differentiation. Several studies have shown that c-Myc activation stimulates sebocyte differentiation [52,53], and therefore we examined c-Myc expression. We found that K14-*Nrg3 *transgenic skin exhibited increased c-Myc expression through most of the basal layer when compared to non-transgenic littermates (Fig. 8E, F). Western blot analysis confirmed an increase in the levels of c-Myc in K14-*Nrg3 *transgenic skin compared to non-transgenic littermates (Fig. 8G)

### Sustained Nrg3 signaling perturbs basement membrane organization

**Figure 9:**
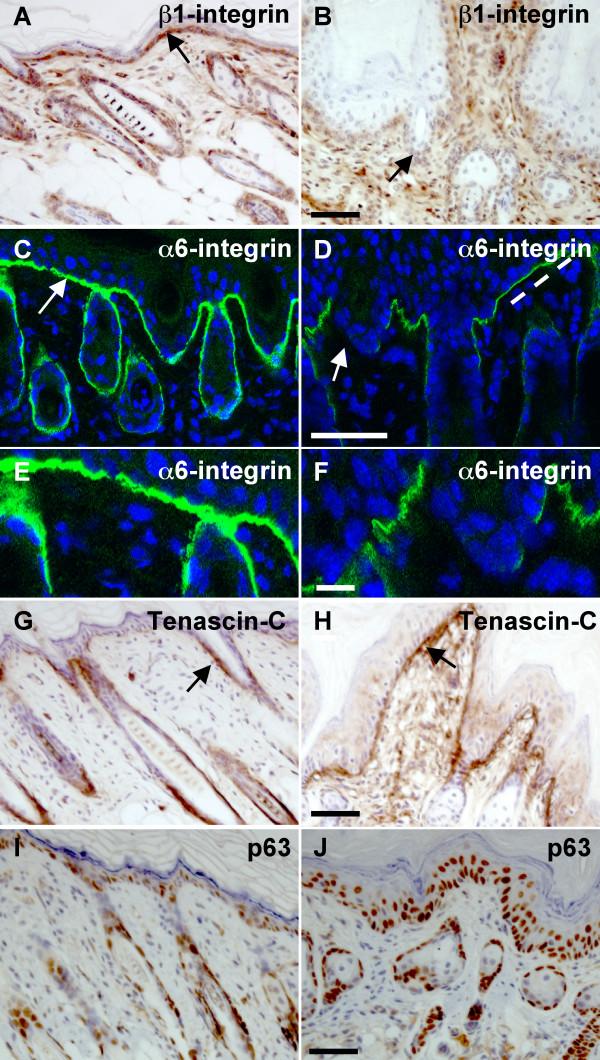
**Nrg3 signaling perturbs the expression of basement membrane components and stem cell markers**. (**A **and **B**) β1-integrin is expressed along the basement membrane (indicated by an arrow) throughout the basal epidermis of non-transgenic littermate (A). β1-integrin expression is reduced in the K14-*Nrg3 *transgenic epidermis (B). (**C **and **D**) α6-integrin is expressed along the basement membrane throughout the basal epidermis of non-transgenic littermate (C and E). α6-integrin expression is reduced in the K14-*Nrg3 *transgenic epidermis (D and F). The basement membrane expression is indicated by an arrow. Some regions of intact basement membrane are present with normal levels of α6-integrin expression, indicated by dashed line, adjacent to regions where α6-integrin expression is reduced. (**G **and **H**) Tenascin-C is expressed along the basement membrane in the vicinity of hair follicles of non-transgenic littermate and is indicated by an arrow (G). Tenascin-C is expressed along the basement membrane throughout the K14-*Nrg3 *transgenic basal epidermis (H). Strong Tenascin-C staining expression is observed at the dermal-epidermal junction and is indicated by an arrow. (**I **and **J**) p63 expression is restricted to epidermal and hair follicle cells with high proliferative potential and is absent in cells undergoing terminal differentiation. p63 expression is restricted to most basal cells and a few suprabasal cells in the IFE and ORS of non-transgenic littermates (I). p63 is expressed throughout most of the basal K14-*Nrg3 *epidermis (J) with expanded expression detected in up to four suprabasal cell layers. The scale bar represents 50 μm except in (E and F) where it represents 12.5 μm.

The extracellular matrix receptor, β1-integrin, is preferentially expressed in many epidermal stem cells and thought to be required to maintain the stem cell compartment. β1-integrin exhibits reduced staining in K14-*Nrg3 *epidermis when compared to non-transgenic littermates (Fig. 9A, B). The hemidesmosomal component, α6-integrin [54], also displayed reduced staining in K14-*Nrg3 *epidermis when compared to non-transgenic littermates (Fig. 9C, D, E, F). Strong adhesion of the basal cells to the underlying basal lamina is provided by the hemidesmosomes. The aberrant staining of cell adhesion markers, α6-integrin and β1-integrin, suggest that partial dissolution of underlying basement membrane has occurred. Tenascin-C, an adhesion-modulating extracellular matrix glycoprotein, is expressed along the basement membrane in the vicinity of hair follicles in normal epidermis (Fig. 9G). There is significant upregulation of Tenascin-C within the basement membrane of K14-*Nrg3 *skin (Fig. 9H).

### Nrg3 activity regulates the fate of epidermal progenitor cells

p63 is normally expressed in most basal epidermal cells and some suprabasal cells and is thought to act to maintain the proliferative potential of stem cells and to mediate epidermal differentiation [55-57]. p63 is expressed in cells with high proliferative potential and is absent in cells undergoing terminal differentiation. We observed expanded expression of p63 which was detected in up to four suprabasal cell layers in K14-*Nrg3 *epidermis. (Fig. 9I, J). A similar distribution of Ki67+ and p63+ cells was observed in the suprabasal layers (compare Figs 9J and 7B). By definition, stem cells rarely cycle and the transit-amplifying cells are actively cycling [58]. This suggests that an increased number of quiescent stem cells are recruited into cell cycle and enter into the transit-amplifying compartment in K14-*Nrg3 *epidermis. Transit-amplifying cells divide several times to produce differentiated cells, which leads to terminal differentiation along various lineages. These results suggest that Nrg3 can influence epidermal stem cell fate decisions and, when ectopically expressed in the basal epidermis, Nrg3 promotes the differentiation of stem cells into epidermal and sebaceous lineages and not along the hair lineage.

## Discussion

We have shown that sustained expression of Nrg3 in the basal epidermis alters the development of epidermal organs including the skin, hair, and mammary glands (Figs 1, 2, 4). Modulation of Nrg3 signaling affects the number of mammary glands by promoting initiation. We have not observed the induction of other ectopic organs in the K14-*Nrg3 *founders aside from mammary glands. However, ectopic Nrg3 did increase the size and alter the shape of most epithelial appendages, which is suggestive of morphogenetic changes. Failure of proper hair differentiation occurred and, in the few cases where hair fibers formed and emerged, only one hair type, zigzag, developed (Fig. 2). Our observation of clusters of hair follicles suggests that in some regions, normal patterning is perturbed (Fig. 5F). In skin from older transgenic mice, the entire hair follicle structure is filled with sebocytes suggesting alterations in cell fate decisions have occurred (Fig. 8D).

K14-*Nrg3 *transgenic mice form extra nipples associated with mammary ducts (Fig. 4). The extra mammary glands develop along and close to the mammary line. Although K14 is expressed in the epidermis from E9.5, it is not upregulated until E14.5 when strong K14 expression is observed in the basal layer of the epidermis, in the outer root sheath and in the bulge cells [37]. K14 upregulation therefore occurs after the mammary placodes have already formed. Our K14-*Nrg3 *transgenics were low copy number integrants and expressed low-levels of the transgene and it is possible that higher levels of expression might elicit other phenotypic effects if it were not lethal. Our previous studies demonstrated both epidermal stratification (the first indication of mammary placode development) and placode formation adjacent to sites of ectopically delivered recombinant Nrg3-Egf domain in mouse embryo explant cultures [2]. The results reported here suggest that, at least in some cases, these ectopic placodes have the ability to complete the entire mammary morphogenetic program.

All four Erbb receptors are expressed in the developing mouse epidermis providing a wide range of signaling possibilities as well as compensatory mechanisms (Fig. 3). Depending on the ligand and the availability of dimerization partners these receptors will then transmit quantitatively or qualitatively different signals in different cell types [59,60]. No significant elevations in Egfr, Erbb2, or Erbb3 were observed as assessed by immunohistochemistry and western blot (Fig. 3 and data not shown). Phosphotyrosine levels were used to assess the activation status of each receptor (Fig. 3). Erbb1 appeared to have slightly increased phosphotyrosine content in the transgenic epidermis. Erbb2, and Erbb3 were active at similar levels in both the transgenic and non-transgenic epidermis. Erbb4 is not normally expressed at significant levels in the postnatal interfollicular epidermis but is expressed throughout the K14-*Nrg3 *transgenic skin (Fig. 3). We observed increased levels of Erbb4 and tyrosine phosphorylation of Erbb4 in the transgenic epidermis when compared to non-transgenic littermates. This is consistent with previous reports that Erbb4 homodimers are the major receptor for Nrg3 [61]. It is likely that cellular mechanisms exist to prevent autocrine signaling of Nrg3-Erbb4, which can be overcome in the K14-*Nrg3 *transgenic model. Our findings suggest that increased Erbb4 signaling in the K14-*Nrg3 *transgenic skin underlies the striking epidermal phenotypes observed in this transgenic model.

Erbb2 is the preferred heterodimerization partner for all of the Erbb receptors [62], so it is likely that Nrg3 elicits its effects through both Erbb2-Erbb4 heterodimers and Erbb4-Erbb4 homodimers (Fig. 3). Normal mammary ductal outgrowth occurs, but lactation fails in mammary glands from both heart-rescued *Erbb4*-null mice (where a myocardial promoter restores *Erbb4 *expression to the heart and overcomes embryonic lethality) and from mice in which *Erbb4 *has been conditionally deleted from the mammary gland [3,4]. Lobular-alveolar units from mice lacking *Erbb4 *in the mammary gland do not express markers of terminal differentiation and exhibit deficient proliferation of the mammary epithelium during pregnancy and at partuition. Erbb4 signaling appears to be required for secretory maturation of the alveoli since milk genes are expressed albeit at reduced levels [3]. Mammary glands from heart-rescued *Erbb2*-null mice display mammary ductal outgrowth defects [7,8]. Nrg1 signaling through Erbb2-Erbb4 heterodimers is required to elicit Stat5 activation and neither Erbb2 nor Erbb4 homodimers alone can activate Stat5 *in vitro *[63]. These results suggest that signaling through Erbb2-Erbb4 heterodimers is likely to be required to achieve terminal differentiation of the mammary gland, but not for ductal outgrowth. *Nrg1*-null, *Erbb2*-null, and *Erbb4*-null mice all display myocardial trabeculation defects which indicates that Nrg1 signaling through Erbb2- Erbb4 heterodimers is necessary for heart development [64-66]. Distinct neural defects including mis-innervation of the hindbrain are found in *Erbb4*-null mice and not in *Erbb2*-null mice, which suggests that this signaling occurs without utilizing Erbb2-Erbb4 heterodimers [64,65]. Erbb4 signaling regulates many other developmental pathways including the migration and differentiation of neuroblasts in the rostral-nasal stream, inhibiting cortical astrogenesis, hypothalamus-mediated reproductive development and function, and blastocyst implantation [67-71]. Signaling through Erbb4 is generally associated with cellular differentiation, particularly in the mammary gland [72]. Erbb4 signaling is likely to be far more complex since active membrane-bound, nuclear and cytosolic forms of Erbb4 exist and have been associated with other cellular processes, including apoptosis and transcriptional repression [73]. Erbb2-Erbb4 heterodimers have higher affinity than Erbb4 homodimers [74]. Signaling through Erbb heterodimers is thought to result in more potent mitogenic response than signalling through Erbb homodimers [75]. Erbb2 can bind to more phosphotyrosine binding proteins, and signaling through Erbb2-Erbb4 heterodimers is generally thought to result in more diverse and distinct outputs than signaling through Erbb4 homodimers [75,76].

We observed unusual *Erbb4 JM *variant profiles in the transgenic epidermis that are distinct from those observed in non-transgenic littermates. Two recent studies have shown that both the CYT-1 isoform and JM-a isoform are overexpressed in the dorsolateral frontal cortex of schizophrenic patients and suggest that dysregulated splice variant expression of *Erbb4 *may underlie the genetic association of Erbb4 with schizophrenia [77,78]. The four different isoforms of Erbb4 have different signaling capabilities and may mediate distinct biological functions [42,43,79]. The contribution of dysregulation of splice-variant specific expression of *Erbb4 *in the K14-*Nrg3 *transgenic skin to the phenotype we observe remains to be elucidated.

The postnatal K14-*Nrg3 *hyperplastic epidermal phenotypes are likely to result from induction of signaling pathways that are not normally continuously activated. Since both c-Myc and CyclinD1 are downstream target genes of β-catenin/Lef [80,81] and the Wnt pathway has well-established roles in both epithelial proliferation [82], cell fate [83], and stem cell maintenance [84], we examined Wnt/β-catenin expression in the K14-*Nrg3 *epidermis. However, we did not detect nuclear β-catenin, altered membranous β-catenin or E-cadherin stain or changes in Lef1 expression in the transgenic epidermis (data not shown).

K14-*Nrg3 *epidermis displays features indicative of perturbed basement membrane organization, including reductions in α6-integrin and β1-integrin expression levels (9). α6-integrin and β1-integrin are cell surface molecules that mediate cell attachment and keratinocyte migration. Both are highly expressed in stem cells and likely to have roles in stem cell maintenance [85,86]. Both human and mouse epidermal stem cells are more adhesive to the extracellular matrix than their daughter cells [86,87]. Reduced β1-integrin levels in human keratinocytes stimulates exit from the stem cell compartment [88]. β1-integrin is required for basement membrane remodelling and for downgrowth of hair follicles [89,90]. Transgenic expression of Nrg3 within the basal epidermis also induces Tenascin-C, an adhesion-modulating extracellular matrix glycoprotein, which is often found at the sites of epithelial-mesenchymal interactions during development and tissue remodeling (Fig. 9) [91,92]. These results suggest that Nrg3 signaling elicits adhesive and extracellular matrix changes that are likely to have significant consequences on progenitor cell behaviour and morphogenetic events.

A number of studies have demonstrated that transgenic expression of c-Myc in the basal cells of the epidermis promotes the differentiation of epidermal stem cells into sebaceous glands. This is a strikingly similar phenotype to our observations in the K14-*Nrg3 *postnatal epidermis [52,53,93]. Consistent with this similarity, c-Myc expression is increased in the basal layer of K14-*Nrg3 *skin (Fig. 8) and changes in K14-*Nrg3* epidermis such as hyperproliferation and increase in cell size (Fig. 2) are consistent with the increased basal expression of c-Myc [94-98]. The extension of expression of p63 into a large number of suprabasal cells in K14-*Nrg3 *skin (Fig. 9) suggests that Nrg3 might cause epidermal cells to exit the stem cell compartment and stimulate the proliferation of transit-amplifying cells, as is also observed in K14-*c-Myc *transgenic skin [53]. Also consistent with c-Myc activation is the decreased α6-integrin and β1-integrin expression observed throughout most of the basal epidermis [52,93]. c-Myc-induced repression of adhesion is thought to stimulate epidermal stem cell differentiation [93]. Our data implies that Nrg3 can alter the proliferation status of basal epidermal cells, most likely, through the induction of c-Myc. However, there are important distinctions between the K14-*Nrg3 *and K14-*c-Myc *models. K14-*c-Myc *mice do form hair which is gradually lost and other epithelial appendage phenotypes have not been reported [52].

These results confirm our previous studies that Nrg3 signaling can regulate epithelial cell fate during mammary gland morphogenesis and suggest Nrg3 can also regulate other pluripotent cell populations in the epidermis. Of particular interest is the ability of Nrg3 to decrease α6-integrin and β1-integrin expression, as this could be the mechanism by which Nrg3 normally elicits epithelial stratification and mammary differentiation at unique sites along the body axes during early mammary morphogenesis. Alterations in basement membrane components are known to have profound effects on progenitor populations perhaps by disrupting the stem cell niche [93,99-101]. The niche is also significantly altered in transgenic MMTV-*c-Myc *mammary epithelia, which show similar changes in stem cell retention and transit-amplifying cell fate decisions as observed in the K14-*c-Myc *epidermis [102]. Conditional deletion of *c-Myc *in the epidermis leads to precocious differentiation and loss of the basal progenitor cells through insufficient expansion [103]. Interestingly, another regulator of Erbb signaling, Lrig, an Egfr antagonist, acts to maintain stem cell quiescence in part by negatively regulating the *c-Myc *promoter [104]. Conditional deletion of *Rac1*, a negative regulator of *c-Myc *leads to rapid depletion of the stem cell compartment and highlights their critical roles in stem cell regulation [105].

We propose that when *Nrg3 *is ectopically expressed in to the postnatal period, as in the K14-*Nrg3 *model, the transit-amplifying cells may differentiate to form the interfollicular epidermis and sebaceous lineages by effectively the same means as proposed by Arnold and Watt [53] and Frye *et al*. [53,93] for the role of c-Myc in the epidermis. In this model, sustained c-Myc activation promotes sebaceous formation at the expense of the hair lineage; sustained Nrg3 expression appears to elicit the same effect. c-Myc expressing human keratinocytes exhibit decreased cell motility [93] which could explain the failure of the hair lineage differentiation in both the K14-*c-Myc *and K14-*Nrg3 *models. Keratinocytes must migrate out from the bulge down to receive the hair inductive signal from the dermal papilla [106,107]. It appears that the sebaceous lineage is conferred on keratinocytes that fail to migrate to the dermal papilla and remain in the bulge.

The exact mechanism by which mammalian epithelia stratifies is not known but p63 is central to this process as stratification fails in its absence [56,108,109]. Recently p63 has been implicated as a key regulator of cellular adhesion and survival of basal cells in the mammary gland and other stratified epithelia [110]. Two models have been proposed (citing either delamination [111,112] or asymmetric cell division [113,114]) for how the stratification of epithelia is elicited. To what degree these contribute to epithelial morphogenesis remains to be ascertained. The onset of stratification during embryogenesis is thought to be elicited by asymmetric cell division [113,114]. Mammary placodes are thought to arise as a result of local cell migration [30] but, initial stages are characterized by stratification [31,32], which is thought to be elicited by basal cell proliferation.

Changes in the adhesive properties of epithelial cells have been described as a general phenomena during the very early stages of bud formation of other organs including the hair follicle, submandibular gland, and mammary gland [115-118]. Both desmosomal and hemidesmosomal expression profiles are downregulated during early mammary bud formation and thought to mediate differences in cell adhesive properties within the forming bud which are subsequently restored during later stages of mammary gland formation [117]. It has been hypothesized that epithelial cells that form the hair follicle respond to the inductive signals from the dermal mesenchyme by changing their cell adhesion properties and that this mediates early morphogenesis [116]. Whether similar changes in adhesive processes are relevant to the very early stages of mammary development (i.e. the initial stratification and placode formation) remain to be determined. The expression of *Nrg3 *in the dermal mesenchyme at the sites where mammary placodes will form prior to their morphological appearance and the ability of Nrg3 to elicit placode formation in explanted embryo cultures would suggest Nrg3 could mediate changes in the adhesive components of the epithelium that lead to formation of the mammary placode.

## Conclusion

Nrg3 is a key regulator of mammary fate. Ectopic expression of Nrg3 induces the formation of supernumerary mammary glands, the expression of c-Myc, and promotes sebaceous gland fate rather than along the hair follicle lineage. Deregulated Nrg3 expression in stem cells reduces the expression of α6-integrin and β1-integrin, both of which are essential for stem cell maintenance and keratinocyte migration. Nrg3 may have roles in promoting mammary lineage commitment and regulation of stem cell maintenance via c-Myc. Further studies of Nrg3 are warranted to discern how mammary epithelial stratification is elicited, particularly with regard to how it coordinates interactions with other signaling pathways and to what degree basal epithelial proliferation contributes to this process.

## Methods

### Generation of K14-*Nrg3 *mice

The cDNA for mouse *Nrg3 *was excised and cloned into the ClaI and SpeI sites of K14-β-globin and an SV40 polyadenylation site was added. The transgene was released from the vector with KpnI and microinjected into fertilized B6 × CBA F1 eggs. Transgenic mice were identified by Southern blotting of tail DNA using a *Nrg3 *cDNA probe.

### Histology, Immunohistochemistry and Immunoflourescence

Skin samples were fixed overnight in 4% paraformaldehyde, formalin, or Carnoy's and then paraffin embedded or embedded in OCT and then frozen immediately in an isopentane bath. Sections (5 μm) were rehydrated, processed with microwave or pressure cooker antigen retrieval if required, and blocked and were incubated with primary antibodies overnight at room temperature.

For immunohistochemistry, peroxidase-labeled polymer (Envision rabbit, Dako, Glostrup, Denmark) was used for detection of primary antibodies raised in rabbits. Biotin-labeled donkey anti-goat IgG antibody (Molecular Probes, Eugene, OR, USA) was used for detection of the goat antibodies using Vector ABC kit (Vector Labs, Burlingame, CA, USA) or Goat Histofine Simple Stain MAX PO (Nicherei, Chou-ku, Tokyo, Japan). Rat Histofine Simple Stain Mouse MAX PO (Nicherei) was used for detection of the rat antibodies. Biotin-labeled rabbit anti-sheep IgG antibody (Vector Labs) was used for detection of the sheep antibodies. MOM kit (Vector Labs) was used for detection of mouse monoclonal antibodies. 3,3' diaminobenzidine was used as chromagen and sections were counterstained with hematoxylin.

The primary antibodies, dilutions, and antigen retrieval conditions used for immunohistochemistry are listed in Additional File 6.

For immunoflourescence, alpha6 integrin antibody (BD Pharmingen) was used on frozen sections at 1:5 dilution followed by secondary anti-rat (Invitrogen, Molecular Probes) antibody conjugated to Alexa Fluor 488 fluorochrome. Nuclei were labeled by using 4'6'-diamidino-2-phenylindole (DAPI). Sections were mounted in Vectashield. Fluorescence samples were examined at room temperature using a TCS SP2 Leica microscope with an Acousto-Optical Beam Splitter.

Oil Red O staining was performed using the standard protocol [119]. Whole mount *in situ *hybridization was performed with *Nrg3 *probe as previously described [2].

### Western blots

Skin samples were homogenized with a Polytron in modified RIPA buffer (50 mM Tris-HCl pH 7.4, 1% Triton X-100, 0.2% sodium deoxycholate, 0.2% SDS, 1 mM EDTA) with protease inhibitors (1 mM PMSF, 5 μg/ml aprotinin, 5 μg/ml leupeptin, 1 mM NaF, 1 mM orthovanadate, 1 mM DTT). Homogenates were analyzed on 10% polyacrylamide gels and transferred to Hybond C extra membranes (GE Healthcare). Filters were incubated with anti-c-Myc (Upstate) overnight at 4°C, washed and incubated with peroxidase-conjugated anti-rabbit IgG (GE Healthcare) and visualized with enhanced chemiluminescence detection kit (ECL, GE Healthcare).

### Immunoprecipitation

Erbb receptor phosphorylation was detected by immunoprecipitating lysates from skin samples with individual receptors and immunoblotting as described [120].

### RT-PCR

*Erbb4 *isoform levels were determined by semiquantitative RT-PCR. cDNAs from transgenic and non-transgenic skin samples were analyzed for *Erbb4 *mRNA variants. A 1.0-μL portion of each cDNA was used as a template in PCR containing 0.3 μM of each primer, 2.25 mM MgCl_2_, 0.5 mM of each dNTP, and 2.5 U of Expand long template enzyme mix (Roche). The amplification conditions were 92°C for 2 min, followed by 35 cycles of 10 sec at 92°C, 30 sec at 57°C (for JM) or 63°C (for CYT), 3 min at 68°C, and a final step of 7 min at 68°C. The following primers were used to amplify the CYT variant of the *Erbb4 *coding region: Erbb4CYTF, GCTGAGGAATATTTGGTCCCCCAG; Erbb4CYTR, AAACATCTCAGCCGTTGCACCCTG. The following primers were used to amplify the JM variant of the *Erbb4 *coding region: Erbb4JMF, GAAATGTCCAGATGGCCTACAGGG; Erbb4JMR, CTTTTTGATGCTCTTTCTTCTGAC. Specificity was verified by sequencing. As a control, *Gapdh *was amplified in parallel. *Gapdh *levels were also determined by semiquantitative RT-PCR.

## Abbreviations

Egfr, EGF receptor; HRG, Heregulin; K14, Keratin14; Nrg3, Neuregulin3.

## Competing interests

The author(s) declares that there are no competing interests.

## Authors' contributions

HP collected tissues, carried out immunohistochemistry, molecular and morphological analysis. OS and SP carried out tissue sectioning and immunohistochemistry. AA participated in the design of the study and helped draft the manuscript. BH conceived and coordinated the study, assisted with experiments, and wrote the manuscript. Figures were prepared by HP and BH. All authors read and approved the final manuscript.

## Supplementary Material

Additional file 1 Shows Nrg3 and Erbb4 expression in the hair follicles. Click here for file

Additional file 2 Shows results from PCR genotyping of founder generation of K14-*Nrg3 *transgenic mice. Click here for file

Additional file 3 Shows the efficiency of transgenesis for males versus females. Click here for file

Additional file 4 Shows the phenotype of a chimeric K14-*Nrg3 *founder mouse. Click here for file

Additional file 5 Shows results of analysis of transgenic skin using immunohistochemistry of epidermal differentiation markers. Click here for file

Additional file 6 Is a list of antigen retrieval methods used for the immunohistochemistry. Click here for file
