# Supplementary material for: Neuregulin3 alters cell fate in the epidermis and mammary gland
Source: BMC Dev Biol. 2007 Sep 19;7:105. doi: 10.1186/1471-213X-7-105 (PMC2110892; doi:10.1186/1471-213X-7-105)
Supplement: Additional file 1 — Shows Nrg3 and Erbb4 expression in the hair follicles. [file 1471-213X-7-105-S1.pdf]

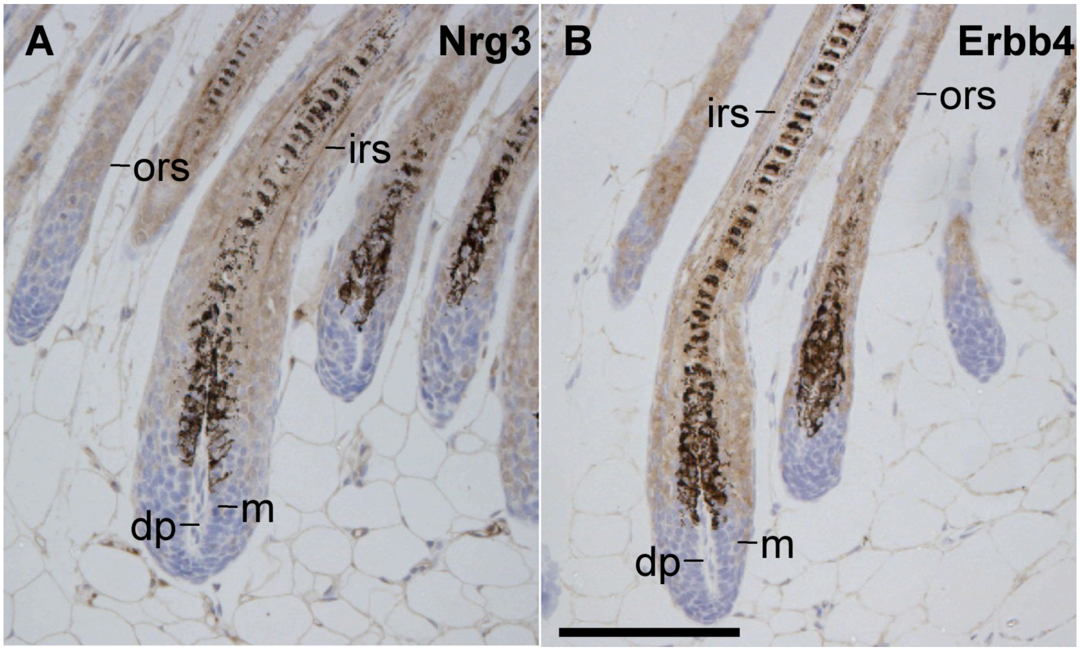

Additional File 1. Immunohistochemical analysis shows the expression of Nrg3 and Erbb4 in hair follicles at P7.

(**A**) Nrg3 is expressed at low to moderate levels in the hair follicles.

(**B**) Erbb4 is expressed at low levels in the hair follicles.

dp, dermal papilla; irs, inner root sheath; m, matrix; ors, outer root sheath.

The scale bar represents 50 μm.
