# Supplementary material for: Neuregulin3 alters cell fate in the epidermis and mammary gland
Source: BMC Dev Biol. 2007 Sep 19;7:105. doi: 10.1186/1471-213X-7-105 (PMC2110892; doi:10.1186/1471-213X-7-105)
Supplement: Additional file 2 — Shows results from PCR genotyping of founder generation of K14-Nrg3 transgenic mice. [file 1471-213X-7-105-S2.pdf]

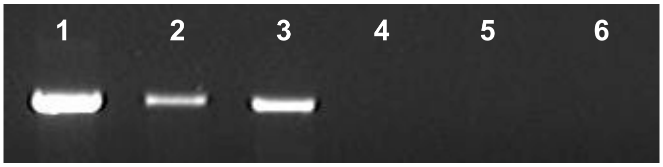

Additional File 2. PCR analysis of K14-*Nrg3* transgenic mice.

Genomic DNA extracted from tail biopsy from each founder were amplified using *Nrg3* specific primers and analyzed by electrophoresis on a 1% agarose gel. Results are shown for three K14-*Nrg3* founders (lanes 1–3), two non-transgenic littermates (lanes 4-5) and a negative control (lane 6). All founder mice displaying the mutant skin phenotype produced a 746 bp product. Littermates with no skin phenotype produced no product. Genomic DNA was also digested with EcoRV and subjected to Southern blot analysis, which produced a 5.3 kb fragment in all transgenic founders (data not shown).
