# Supplementary material for: Neuregulin3 alters cell fate in the epidermis and mammary gland
Source: BMC Dev Biol. 2007 Sep 19;7:105. doi: 10.1186/1471-213X-7-105 (PMC2110892; doi:10.1186/1471-213X-7-105)
Supplement: Additional file 3 — Shows the efficiency of transgenesis for males versus females. [file 1471-213X-7-105-S3.pdf]

	Males	Females
number of transgenic pups	6	4
total number of pups	86	63
efficiency (%)	5.4	2.7

Additional File 3. Efficiency of trangenesis of K14-*Nrg3* transgenic mice.
