# Supplementary material for: Neuregulin3 alters cell fate in the epidermis and mammary gland
Source: BMC Dev Biol. 2007 Sep 19;7:105. doi: 10.1186/1471-213X-7-105 (PMC2110892; doi:10.1186/1471-213X-7-105)
Supplement: Additional file 4 — Shows the phenotype of a chimeric K14-Nrg3 founder mouse. [file 1471-213X-7-105-S4.pdf]

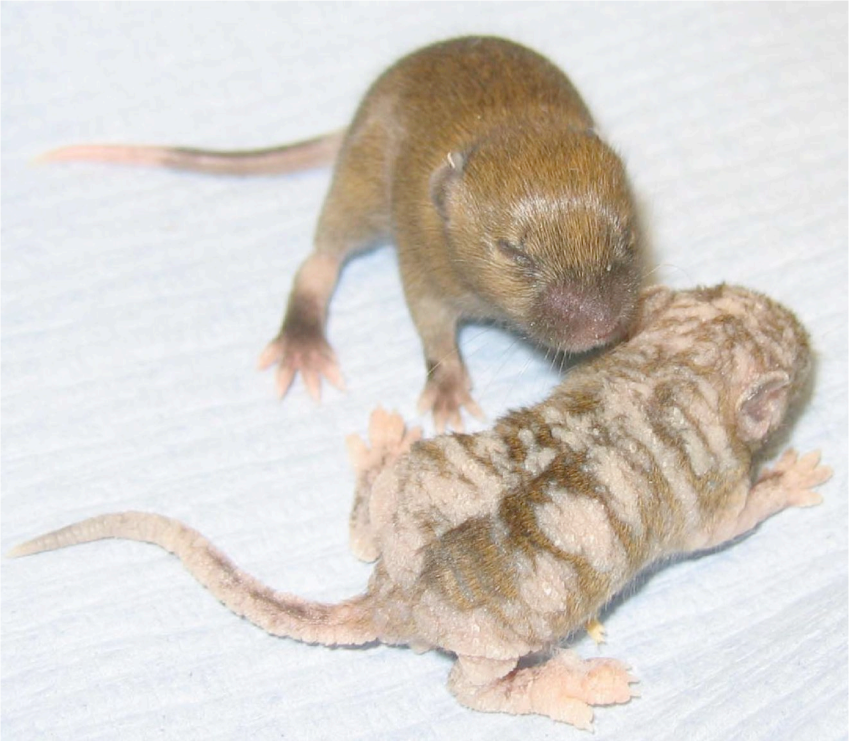

Additional File 4. Postnatal phenotype of a K14-*Nrg3* chimeric founder mouse.

Phenotype of one K14-*Nrg3* founder at P10. The mutant mouse shows hairless, thick, pale and wrinkled skin on about one half of the body surface, which is consistent with the transgene integrating at the two-cell stage. A non-transgenic littermate is on the top left.
