# Supplementary material for: Neuregulin3 alters cell fate in the epidermis and mammary gland
Source: BMC Dev Biol. 2007 Sep 19;7:105. doi: 10.1186/1471-213X-7-105 (PMC2110892; doi:10.1186/1471-213X-7-105)
Supplement: Additional file 5 — Shows results of analysis of transgenic skin using immunohistochemistry of epidermal differentiation markers. [file 1471-213X-7-105-S5.pdf]

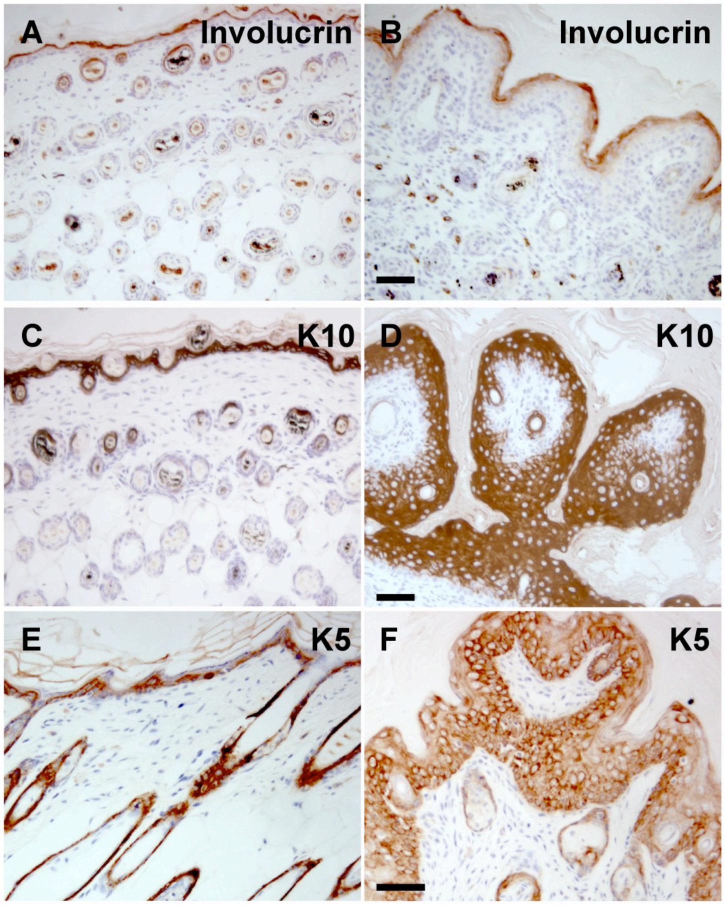

Additional File 5. Immunohistochemistry using epidermal differentiation markers. Immunohistochemistry of sections of dorsal skin from transgenic mice and non-transgenic littermates at P7 with antibodies to the proteins indicated.

(**A** and **B**) Expression of Involucrin, a terminal differentiation marker, is also expressed in the granular and cornified layers of non-transgenic (A) and K14-*Nrg3* transgenic (B) skin.

(**C** and **D**) Expression of K10, a marker for committed early differentiating cells, is expressed in the suprabasal layer of non-transgenic (C) and K14-*Nrg3* transgenic (D) skin.

(**E** and **F**) Expression of K5, a marker for basal proliferating keratinocytes, is expressed in the basal layer of non-transgenic skin and the ORS of the hair follicles (E) and expressed at high levels throughout the basal and suprabasal layers of K14-*Nrg3* transgenic skin and ORS of the hair follicles (F). The scale bar represents 50 μm.
