# Supplementary material for: Neuregulin3 alters cell fate in the epidermis and mammary gland
Source: BMC Dev Biol. 2007 Sep 19;7:105. doi: 10.1186/1471-213X-7-105 (PMC2110892; doi:10.1186/1471-213X-7-105)
Supplement: Additional file 6 — Is a list of antigen retrieval methods used for the immunohistochemistry. [file 1471-213X-7-105-S6.pdf]

Antibody	Raised in	Fix	Antigen retrieval	Dilution	Manufacturer
β1-integrin	Rat	Carnoys	none	1:500	Chemicon
c-Myc (Ab-3)	Mouse	4% PFA	PC	1:10	Calbiochem
CyclinD1 (Clone SP4)	Rabbit	Formalin	2 min PC in 10 mM EDTA pH8.0	1:50	Labvision
E-cadherin	Mouse	Carnoys	none	1:500	BD Transduction Laboratories
EGFR	Sheep	Frozen	none	1:25	Upstate
Erbb2 (C-18)	Rabbit	Frozen	none	1:100	Santa Cruz
Erbb3 (C-17)	Rabbit	Carnoys	none	1:50	Santa Cruz
Erbb4 (C-18)	Rabbit	4% PFA	MW	1:300	Santa Cruz
Filaggrin	Rabbit	Carnoys	none	1:6000	Covance
Involucrin	Rabbit	Carnoys	none	1:7500	Covance
K1	Rabbit	4% PFA	none	1:12000	Covance
K5	Rabbit	4% PFA	none	1:7000	Covance
K10	Rabbit	Carnoys	none	1:2500	Covance
K14	Rabbit	4% PFA	MW	1:3000	Covance
K15	Mouse	Carnoys	none	1:350	Labvision
K2e	Mouse	PFA	MW	1:100	Abcam
Ki67 (Clone Tec3)	Rat	Formalin	18 mins MW in Dako solution pH6.0	1:125	Dako
Nrg3	Rabbit	Formalin	MW	1:50	Abgent
p63	Mouse	Formalin	PC	1:200 1hour at RT	Santa Cruz
Tenascin-C	Rabbit	Formalin	none	1:20	IBL Co., Ltd

Additional File 6. Immunohistochemistry methods.

MW – 18 minutes in microwave in citrate buffer pH 6.0, PC – pressure cooking for 2 minutes in citrate buffer pH 6.0. All antibodies used for immunohistochemistry were incubated overnight at room temperature unless otherwise stated.
